# Calculating hospital length of stay using the Hospital Episode Statistics; a comparison of methodologies

**DOI:** 10.1186/s12913-017-2295-z

**Published:** 2017-05-12

**Authors:** John Busby, Sarah Purdy, William Hollingworth

**Affiliations:** 1 Postdoctoral Research Fellow, Centre for Public Health, Queen’s University Belfast, Belfast, UK BT12 6BA; 2 School of Social and Community Medicine, University of Bristol, BS8 2PS Bristol, UK

**Keywords:** Length of stay, Hospitals, Methods

## Abstract

**Background:**

Accurate calculation of hospital length of stay (LOS) from the English Hospital Episode Statistics (HES) is important for a wide range of audit and research purposes. The two methodologies which are commonly used to achieve this differ in their accuracy and complexity. We compare these methods and make recommendations on when each is most appropriate.

**Methods:**

We calculated LOS using continuous inpatient spells (CIPS), which link care spanning across multiple hospitals, and spells, which do not, for six conditions with short (dyspepsia or other stomach function, ENT infection), medium (dehydration and gastroenteritis, perforated or bleeding ulcer), and long (stroke, fractured proximal femur) average LOS. We examined how inter-area comparisons (i.e. benchmarking) and temporal trends differed. We defined a classification system for spells and explored the causes of differences.

**Results:**

Stroke LOS was 16.5 days using CIPS but 24% (95% CI: 23, 24) lower, at 12.6 days, using spells. Smaller differences existed for shorter-LOS conditions including dehydration and gastroenteritis (4.5 vs. 4.2 days) and ENT infection (0.9 vs. 0.8 days). Typical patient pathways differed markedly between areas and have evolved over time. One area had the third shortest stroke LOS (out of 151) using spells but the fourth longest using CIPS. These issues were most profound for stroke and fractured proximal femur, as patients were frequently transferred to a separate hospital for rehabilitation, however important disparities also existed for conditions with simpler secondary care pathways (e.g. ENT infections, dehydration and gastroenteritis).

**Conclusions:**

Spell-based LOS is widely used by researchers and national reporting organisations, including the Health and Social Care Information Centre, however it can substantially underestimate the time patients spend in hospital. A widespread shift to a CIPS methodology is required to improve the quality of LOS estimates and the robustness of research and benchmarking findings. This is vital when investigating clinical areas with typically long, complex patient pathways. Researchers should ensure that their LOS calculation methodology is fully described and explicitly acknowledge weaknesses when appropriate.

## Background

Within the UK, hospital bed capacity has come under increasing pressure from the dual threat of growing demand within emergency departments [1] and increasing discharge delays [2]. Reductions to hospital length of stay (LOS) could release pressure on beds, provide a timely boost to deteriorating hospital finances [3], and improve patient outcomes (e.g. reduced infections [4]). Benchmarking, where hospitals or regions are compared to identify opportunities for LOS reductions, could be undermined by inaccuracies in the way LOS is commonly calculated and reported. Accurate LOS calculations are crucial for a variety of other audit and research purposes including forecasting patient flow, designing interventions to reduce discharge delays, and evaluating policy impact.

Within England, LOS measurement is primarily driven by the way hospital care is reported [5]. Inpatient treatment is recorded by hospitals, collated by the Health and Social Care Information Centre (HSCIC), and released as part of the Hospital Episode Statistics (HES). HES data are widely used by publicly-funded and commercial organisations, including the National Health Service (NHS), to better understand and improve hospital care. HES are recorded at the finished consultant episode (FCE) level, which represents the time spent under the care of a single consultant. These are frequently joined together to create spells [6–10], the time spent within a single hospital (which may include multiple FCEs), or continuous inpatient spells (CIPS) [11–15], the entire period of inpatient care (which may include spells at multiple hospitals).

FCEs and spells are susceptible to vagaries in the way hospitals organise their care, and in particular their propensity to transfer patients between consultants or to new hospitals. Theoretically, CIPS overcome these limitations and provide a more reliable measure of LOS, however creating these requires episode-level data, substantial computational power and experienced analysts. For this reason organisations often default to a spell-based analysis [5], however the impact of this decision on study findings remains unclear. An improved understanding of the bias of spell-based LOS could increase the quality of data provided to policymakers, lead to more robust decisions, and improved patient outcomes.

In this paper we empirically investigate the magnitude of differences between using a CIPS- and spell-based methodology when calculating LOS nationally, benchmarking across areas, and investigating temporal trends. We define a classification system for spells and use this to explore the causes of differences.

## Methods

### Data

This study was completed as part of a wider programme of work investigating geographic variation in unplanned ambulatory care sensitive condition (ACSC) admission rates. ACSCs are those where admission can potentially be avoided through improved community or primary care [16]. Our original study included 28 common ACSCs however, for simplicity, we focussed this study on a subset of six. We selected two conditions with a short LOS (dyspepsia or other stomach function, ENT infection), medium LOS (dehydration and gastroenteritis, perforated or bleeding ulcer) and long LOS (stroke, fractured proximal femur). We identified admissions for each condition using ICD-10 diagnosis codes from previous work (Appendix) [16].

We used the HES admitted patient care dataset to identify admissions between 1^st^ April 2007 and 31^st^ March 2012 [17]. We joined FCEs to create spells, and then joined spells to create CIPSs using a unique patient identifier. CIPS spanning over the extract end date (31^st^ March 2012) were censored or omitted from the HES dataset entirely. Therefore we excluded all episodes in the 90 days prior to the extract end date and all CIPS (and their constituent episodes and spells) lasting more than 90 days. Stays censored by death were also excluded as they do not represent a complete hospital stay. Our analysis compared LOS across primary care trusts (PCTs). Until 2013 there were 151 PCTs in England which were responsible for commissioning most of the healthcare for their populations. PCTs have now been replaced by clinical commissioning groups (CCGs) which perform a broadly similar role but place an increased emphasis on the role of general practitioners.

### Classifying spells

Patient pathways can be complicated. To better understand the reasons for differences between a CIPS- and spell-based analyses we developed a classification system which categorised spells into four mutually exclusive and exhaustive categories. For ease of understanding we focus on a calculation of LOS for unplanned stroke admissions below, however the arguments are identical for other conditions.

**Admission spell:** The first spell within a CIPS. It encompasses the time between a patient being first admitted to hospital for unplanned stroke care until they are either discharged or transferred to another hospital.

**Transfer spell:** A subsequent spell after a patient is transferred to a different hospital to receive unplanned stroke care.

**Rehabilitation spell:** A subsequent spell after a patient is transferred to a different hospital to receive planned stroke care.

**New condition spell:** A subsequent spell after a patient is transferred to a different hospital to receive treatment for a non-stroke problem.

**Fig. 1:**
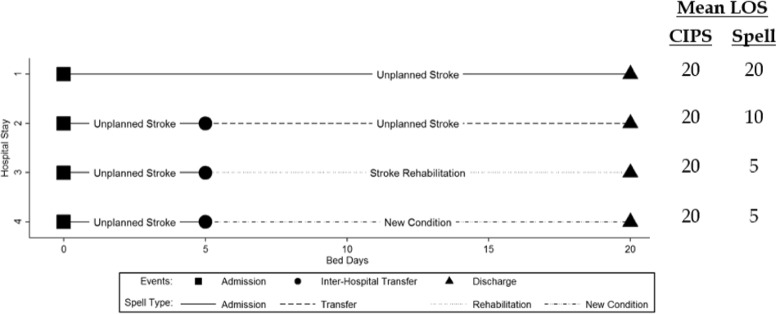
Comparison of mean LOS for stroke care using CIPS and spells

For example a patient with acute stroke might be first admitted to a local hospital (admission spell), transferred to a stroke unit for acute treatment (transfer spell), transferred back to a local hospital for rehabilitation (rehabilitation spell) and have an adverse event (e.g. fall) requiring transfer to an acute hospital (new condition spell). Figure 1 displays these graphically and highlights the major weaknesses of a spell-based methodology. Among four hospital stays lasting an identical amount of time (20 days), the estimated mean unplanned LOS using spell-based methods differs substantially depending on the patient’s pathway. As there is no linkage across hospitals in a spell-based analysis, time spent in ‘rehabilitation’ and ‘new condition’ spells is excluded entirely. Therefore patients admitted for unplanned stroke care, and then transferred to another hospital for rehabilitation or non-stroke care, may have only a fraction of their total hospital stay included in the LOS calculation (Spell 3 & 4, Fig. 1). Furthermore, whilst time spent in ‘transfer’ spells is included, these are treated as new admissions causing the mean LOS to be decreased by at least one half (Spell 2, Fig. 1). LOS is accurately measured when using CIPS regardless of the patient’s pathway, and is therefore regarded as the ‘gold-standard’ methodology.

### Analysis

We calculated the number of unplanned hospital stays, total bed days and mean LOS under a CIPS- and spell-based methodology for each year of the study. We calculated the percentage difference between a CIPS and spell analysis for each of these metrics. Using both methods, we also ranked PCTs from highest (longest mean LOS) to lowest (shortest mean LOS). We calculated the median and maximum absolute difference in rankings. To better understand the differences between a CIPS and spell analysis we calculated the proportion of hospital time spent in ‘admission’, ‘transfer’, ‘rehabilitation’ and ‘new condition’ spells at both the national and PCT level. As preliminary analysis revealed that time spent in rehabilitation spells was the most important driver of differences we explored how this differed across PCTs and evolved over time. We estimated 95% confidence intervals using non-parametric bootstrapping.

## Results

### National

Stroke and fractured proximal femur mean LOS was 23.8% (95% CI: 23.2, 24.3) and 19.3% (95% CI: 18.8, 19.8) shorter when calculated using spells rather than CIPS (Table 1). Much smaller, although still important, differences of between 3.7% and 5.5% were found for other conditions. Differences mainly resulted from a lower number of bed days in the spell analysis and, more specifically, the exclusion of a substantial amount of time spent in rehabilitation (Table 2). For example, the 140,712 stroke bed days (19.6% of CIP total) spent in rehabilitation accounted for the vast majority (94%) of the 150,345 bed day disparity between and a CIPS- and spell-based analysis. Double counting of hospital admissions played a relatively minor role in driving differences in mean LOS as ‘transfer’ spells were rare across all conditions.

**Table 1:** Comparison of admission counts, total bed days and mean LOS for a CIPS and spell analyses

Condition	Hospital stays			Total bed days (1,000s)			Mean LOS		
	CIPS	Spell	% Difference	CIPS	Spell	% Difference	CIPS	Spell	% Difference (95% CI)
Stroke	43,526	45,167	3.8	719	569	-20.9	16.5	12.6	-23.8 (-24.3,-23.2)
Fractured proximal femur	38,093	38,930	2.2	845	697	-17.5	22.2	17.9	-19.3 (-19.8,-18.8)
Perforated or bleeding ulcer	53,958	54,157	0.4	246	233	-5.2	4.6	4.3	-5.5 (-6.0,-5.0)
Dehydration and gastroenteritis	87,270	87,506	0.3	391	371	-5.2	4.5	4.2	-5.5 (-5.9,-5.1)
Dyspepsia or other stomach function	14,243	14,256	0.1	18	18	-4.9	1.3	1.2	-5.0 (-7.0,-3.5)
ENT infection	57,402	57,523	0.2	49	48	-3.5	0.9	0.8	-3.7 (-4.4,-3.0)

**Table 2:** Distribution of hospital stay across spell types

Condition	Days (1,000s) and percentage (brackets) spent in spell type				Total bed days (1,000s)
	Admission	Transfer	Rehabilitation	New condition	
Stroke	537 (74.6)	32 (4.5)	141 (19.6)	10 (1.3)	719
Fractured proximal femur	678 (80.2)	18 (2.2)	138 (16.4)	10 (1.2)	845
Perforated or bleeding ulcer	232 (94.3)	2 (0.6)	9 (3.6)	4 (1.5)	246
Dehydration and gastroenteritis	369 (94.4)	2 (0.4)	15 (3.8)	5 (1.3)	391
Dyspepsia or other stomach function	17 (95.1)	0 (0.2)	0 (2.6)	0 (2.0)	18
ENT infection	47 (96.2)	0 (0.6)	1 (1.9)	1 (1.4)	49

### Benchmarking

**Fig. 2:**
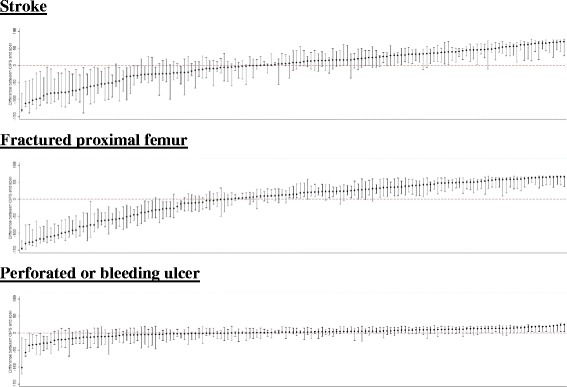
Comparison of PCT ranking in the CIPS and spell analysis. Circles represent the difference in LOS rank between a CIPS and spell analysis for each PCT. Vertical lines represent 95% confidence intervals. Dashed red line represents equality in ranks between the CIPS and spell analysis

**Fig. 3:**
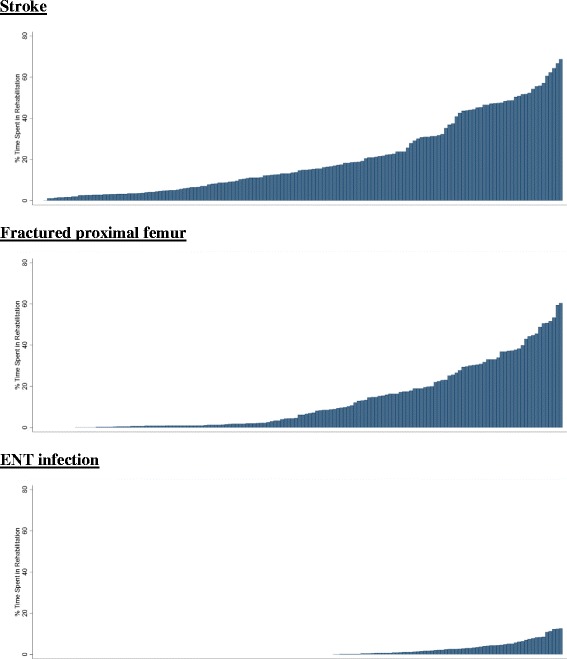
Proportion of rehabilitation time across PCTs. Bars represent the proportion of time spent within rehabilitation spells for a given PCT

For fractured proximal femur and stroke, conditions with the longest LOS, there was little accord in the rankings of PCTs when using a CIPS- or spell-based methodology to calculate LOS (Fig. 2). The median difference for fractured proximal femur was 40 ranks, with extremely large disparities for individual PCTs (Fig. 2). For example, one PCT (PCT A) had the third highest rank (longest mean LOS) for fractured proximal femur when using a CIPS methodology yet the forth lowest (shortest LOS) under a spell framework representing a gulf of 145 (95% CI: 122, 148) ranks. Differences between rankings based on CIPS and spells were generally driven by extreme variability in the proportion of time spent in rehabilitation spells across PCTs (Fig. 3). In PCT A 61% of fractured proximal femur hospital stays were spent in rehabilitation meaning that a spell-based analysis provided an extreme underestimate of mean LOS compared to using CIPS (10.6 days vs. 28.6 days). In contrast, there were no transfers recorded among patients admitted for fractured proximal femur in another PCT (PCT B) meaning that LOS was identical regardless of how it was calculated. The median difference was seven ranks or less for the other conditions included in our study, however important differences in excess of 30 ranks, and up to 102 ranks for perforated or bleeding ulcer, still existed for some PCTs (Fig. 2).

### Temporal trends

**Fig. 4:**
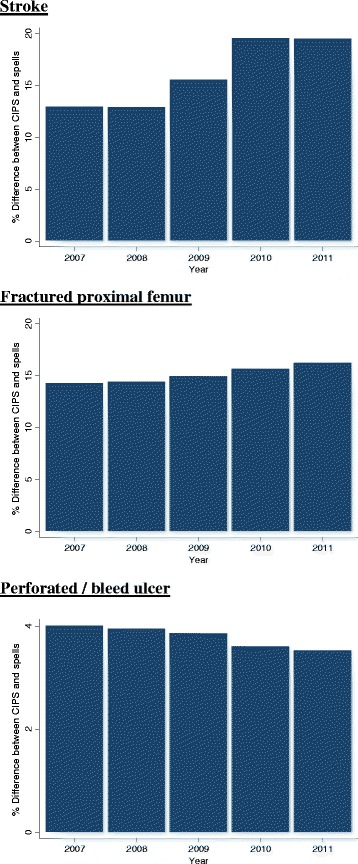
Temporal trend in mean LOS under a CIPS and spell analysis. Bars represent the difference between a CIPS and spell analysis in each year

In general, the discrepancy between a CIPS- and spell-based analyses increased over the study period (Fig. 4). For example, the difference in stroke LOS was 16.4% (95% CI: 15.9, 16.9) during 2007/8 and 23.8% (95% CI: 23.1, 24.3) during 2011/12. Similarly, the discrepancy for fractured proximal femur was 16.2% (95% CI: 15.9, 16.6) during 2007/8 and increased to 19.3% (95% CI: 19.0, 19.7) during 2011/12. In both cases these disparities where driven by a sharp increase in the number of rehabilitation spells. This was most pronounced among stroke patients where the proportion of time spent in rehabilitation increased by over 50% during the study period from 13.0% in 2007/8 to 19.6% in 2011/12. The difference between a CIPS- and spell-based analyses was relatively consistent across time for other conditions where patients were are typically treated within a single hospital (e.g. perforated or bleeding ulcer).

## Discussion

### Statement of principal findings

Measuring length of stay using spells can lead to substantial underestimates of nearly 25% for some conditions. The typical patient pathway often differs between areas. Under a spell-based analysis this can impair benchmarking and lead regions to appear efficient simply because they transfer a large proportion of patients for rehabilitation. In general, the time spent in rehabilitation spells has increased over time which could undermine examination of temporal trends in LOS under a spell-based analysis. Each of these issues were most profound for stroke and fractured proximal femur, as patients were frequently transferred to a separate hospital for rehabilitation, however important disparities also existed for conditions with simpler pathways (e.g. ENT infections, dehydration and gastroenteritis).

### Strengths and weaknesses

This analysis addresses an important, but often overlooked, methodological issue when using the HES dataset to calculate LOS nationally, compare between regions, or investigate temporal trends. By including a diverse range of conditions we have identified the circumstances under which these biases are largest, and when they are perhaps tolerable. We have used bootstrapping methods to calculate sampling distributions around key parameters (e.g. ranking of PCTs) which provides an objective measure of uncertainty.

The main weakness in our study lies in its potential lack of generalisability beyond those using the HES dataset; nevertheless it seems likely that similar issues will exist in any country where administrative data is collected within hospitals and used to guide decision making. The HES dataset is widely used for research and audit purposes [5] meaning that our findings have extremely important implications for NHS policymaking. Our decision to exclude spells censored by death may have introduced a small bias into our results. It is possible that more advanced competing-risk survival models could be used to overcome this [18]. The spell classifications used within our analysis may be too simplistic to differentiate between the myriad of pathways a patient my take during a hospital stay. Future investigation into the causes and consequences of variable hospital pathways may require a more comprehensive system. For example, it may be useful to delineate ‘new condition’ spells related to medical error from those which are unpreventable.

### Comparison with other studies

To our knowledge this is the first study to compare LOS using CIPS and spells. A previous study [19] found substantial differences in admission counts when they were calculated using FCEs and spells. Although that study did not investigate LOS, it seems highly likely that such analysis would have demonstrated disparities in LOS. In agreement with our findings, one study has highlighted vast differences among hospitals in the use of rehabilitation centres for patients admitted with hip fracture [20].

### Implications for clinicians and policymakers

Accurate calculation of LOS is extremely important for a wide range of audit and research purposes. Benchmarking has been identified as a key tool to drive productivity savings in the National Health Service [21], however our analysis demonstrates this can be completely undermined when using spell LOS. This could severely limit the ability of NHS organisations to identify and act on improvement opportunities. Cross-sectional studies investigating the effect of patient or hospital characteristics on LOS have been commonly used to identify the most important drivers of LOS, and develop interventions to reduce discharge delays. However these factors (e.g. condition volume [22], clinical guidelines [23]) could appear to be strongly associated with spell LOS, when a relationship doesn’t actually exist with the total time spent in hospital, if they are correlated with the probability of hospital transfer. Similarly, before and after studies have been commonly utilised to investigate the effect of healthcare policies (e.g. payment by results [10], centralisation of stroke care [11]) on LOS however, when using a spell-based methodology, changes in LOS could be due to evolving patient pathways (e.g. more transfers for stroke rehabilitation) rather than any true difference in the time spent within hospital. It is unlikely that the deficiencies of a spell-based analysis could be overcome by statistical adjustment, as the causes of differing hospital pathways are likely to be complex and, in many cases, intangible. Our analysis empirically describes the potential bias of a spell-based analysis for the first time, and should provide a stimulus for improved methodological rigour.

Despite the pitfalls of calculating LOS using spells, this methodology is widely employed by NHS organisations and academic researchers. The HSCIC, which is the national provider of data to analysts and commissioners, presents national-level LOS using spells [24]. Perhaps of even greater concern, given the results in our study, is that several NHS benchmarking tools including the NHS Better Care, Better Value Indicators [25], NHS Compendium of Information [26], and the RightCare Atlas of Variation [27] base at least some of their outputs on spell LOS. Inaccurate benchmarking analysis could lead to vital improvement opportunities being missed, or costly investigations being launched to solve problems that don’t actually exist. The academic literature also contains many studies using spell LOS which could undermine their conclusions [6–10]. For example, a study finding an association between the introduction of payment by results and reduced spell LOS [10] might be confounded by an increasing proportion of hospital transfers over time. Similarly, another study finding inter-hospital variation in four common types of surgery may simply reflect differences in patient pathways rather than true disparities in the time spent in hospital [6]. Several studies did not provide sufficient detail on the methodology used to calculate LOS [28–30] which prevents readers from determining the robustness of their results.

Our results highlight the need for a step change in how LOS is calculated and reported. National data providers, such as the HSCIC, have sufficient resources to routinely report CIPS-based LOS and should switch to this methodology. Higher quality data could lead to more robust decisions and improved patient outcomes. Similarly, publishers of LOS benchmarking tools should ensure these are based on CIPS as spell-based comparisons are unreliable, even for conditions where care is typically provided by a single hospital. At the very least, spell-based LOS comparisons should explicitly acknowledge the weaknesses of this approach and advise caution when interpreting the results. It is perhaps understandable that small research teams with limited resources sometimes forgo the complex procedure of creating CIPS, and instead opt to use spell LOS. Our results suggest that this may be defensible providing they do not compare across areas and are solely interested in clinical areas where care is typically provided within a single hospital. Such analyses should always be accompanied by a report on the proportion of spells which end with a hospital transfer. However, CIPS-based analysis is always preferable and should be conducted when possible.

## Conclusions

Accurate calculation of LOS is extremely important for a wide range of audit and research purposes. However commonly used spell-based methodologies may omit important parts of the patient’s pathway and undermine analyses aiming to calculate LOS nationally, benchmark across areas, and investigate temporal trends. National reporting organisations and researchers should calculate LOS using CIPS, particularly when investigating clinical areas with complex patient pathways, or conducting benchmarking. Researchers should ensure that their LOS calculation methodology is fully described and explicitly acknowledge weaknesses where appropriate. Future investigation into the causes and consequences of variable hospital pathways is required to understand its impact on healthcare costs and patient outcomes.

## Appendix

Included ACSCs and ICD-10 codes used to define them. List of ICD-10 codes used to identify admissions for each condition within the analysis.

## Abbreviations

ACSC: Ambulatory care sensitive condition
CCG: Clinical commissioning group
CIPS: Continuous inpatient spells
ENT: Ear, nose and throat
FCE: Finished consultant episode
HES: Hospital episode statistics
HSCIC: Health and social care information centre
LOS: Length of stay
NHS: National health service
PCT: Primary care trust
